# Adhesion of yeast cells to different porous supports, stability of cell-carrier systems and formation of volatile by-products

**DOI:** 10.1007/s11274-012-1151-x

**Published:** 2012-08-19

**Authors:** Dorota Kregiel, Joanna Berlowska, Wojciech Ambroziak

**Affiliations:** Institute of Fermentation Technology and Microbiology, Technical University of Lodz, Wolczanska 171/173, 90-924 Lodz, Poland

**Keywords:** Yeasts, Adhesion, Chamotte, Hydroxylapatite, Fermentation

## Abstract

The aim of our research was to study how the conditions of immobilization influence cell attachment to two different ceramic surfaces: hydroxylapatite and chamotte tablets. Three fermentative yeast strains, namely brewery TT, B4 (ale, lager) and distillery Bc15a strains belonging to *Saccharomyces* spp., and one strain of *Debaryomyces occidentalis* Y500/5 of weak fermentative nature, but with high amylolytic activity due to extracellular α-amylase and glucoamylase, were used in this study. Different media, including cell starvation, were applied for immobilization of yeast strains as well as different phases of cell growth. Immobilization of selected yeasts on a hydroxylapatite carrier was rather weak. However, when incubation of starved yeast cells was conducted in the minimal medium supplemented by calcium carbonate, the scale of immobilization after 24 h was higher, especially for the *D. occidentalis* strain. Adhesion to hydroxylapatite carriers in wort broth was of reversible character and better results of adhesion were observed in the case of another ceramic carrier-chamotte. The number of immobilized cells was about 10^6^–10^7^ per tablet and cell adhesion was stable during the whole fermentation process. The comparison of the volatile products that were formed during fermentation did not show any significant qualitative and quantitative differences between the free and the immobilized cells. This is the first time when a cheap, porous chamotte surface has been applied to yeast adhesion and fermentation processes.

## Introduction

The previous studies on yeast adhesion mainly concentrated on the pathogenic species *Candida albicans* (Denaro et al. 1995; Jain et al. 2010; Jin et al. 2004; Umazume et al. 1995). Nowadays, the research is also focused on the biotechnological application of immobilized industrial yeast strains in different biotechnological processes. The available literature presents numerous immobilization methods suitable for the production of various beverages (Kourkoutas et al. 2004; Nedovic et al. 2011; Verbelen et al. 2006). Nevertheless, the successful use of the immobilized cell systems for industrial scale processes is rather rare—the unbalanced pattern of the final fermentation product may reduce customer’s acceptability (Willaert and Nedovic 2006).

It is evident that the chemical composition of the solid carrier and the surface topography influence cell adhesion. In many cases, substantial roughness/porosity of the carrier promotes cell attachment to the surfaces because of increased surface area available for binding. If the surface consists of deep channels, cell adhesion is usually more intensive (Edwards and Rutenberg 2001; Hou et al. 2011; Pereira et al. 2000). Similarly, if the cell and the surface have opposite charges, the electrostatic forces are more attractive. However, in case of microbial cells and potential adhesion surfaces, the surface charges are usually negative, which leads to electrostatic repulsions. The repulsion forces decrease with an increase of some factors, such as the ionic strength of the culture medium. The ionic strength determines the thickness of the electrical double layer, which has a direct influence on electrostatic interactions established in the adhesion process (Buck and Andrews 1999; Hermansson 1999; Henriques et al. 2004; Yin et al. 2002). Increased cell surface hydrophobicity can facilitate surface approaching and triggering specific forces responsible for adhesion (Liu et al. 2004; Lorite et al. 2011; White and Walker 2011).

Yeast cell immobilization can influence the production of the main products as well as the by-products. Ethanol, carbon dioxide and other flavor-active compounds develop during the fermentation process. Their production is closely related to the metabolic activity and physiological state of immobilized yeast cells (Brányik et al. 2006, 2008).

The immobilized cell technology offers considerable advantages for different fermentation industries. Effective adhesion of yeasts on different solid carriers, e.g. porous glass, polyurethane foam, ceramics or even spent grains can be used in many fermentation processes, including beer fermentation (Brányik et al. 2006; Verbelen et al. 2006; White and Walker 2011; Willaert and Nedovic 2006). Moreover, immobilized yeast cells may be a valuable source of numerous enzymes useful in biotechnological processes. *Debaryomyces occidentalis* is of biotechnological interest primarily because of its ability to produce different hydrolytic enzymes, especially its active amylolytic complex. This system is capable of degrading different starch sources from barley, corn or wheat. The ability of *Debaryomyces* spp. to tolerate extreme stress could be additionally advantageous in the implementation of low-cost fermentation processes (Johnson and Echavarri-Erasun 2011).

The results of the previous studies stimulated our research. The main aim of this study was to compare the adhesion abilities of industrially relevant yeast strains—brewery (ale and lager), distillery yeasts belonging to *Saccharomyces* ssp. and an amylolytic strain of *Debaryomyces* *occidentalis*. In the study, we used two kinds of ceramic carriers: hydroxylapatite (Hap) and chamotte. Additionally, the stability of the cell—carrier systems and production of selected volatile by-products by the tested yeast strains were investigated. Hap has different biomedical applications. It was also used by White and Walker (2011) for the purpose of yeast adhesion. Nevertheless, this work is the first one that has described the application of another interesting material-cheap, porous chamotte as a carrier for yeast cell adhesion and the fermentation processes.

## Materials and methods

### Carriers

**Fig. 1:**
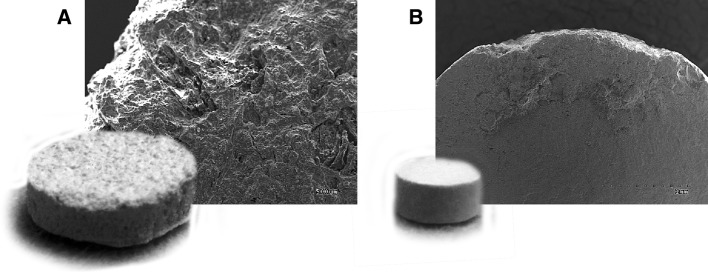
Two ceramic carriers: **a** chamotte, **b** hydroxylapatite

Two kinds of ceramic carriers-hydroxylapatite [Ca_5_(PO_4_)_3_(OH)] and chamotte [mainly Al_2_O_3_ (36 %) with SiO_2_ (58 %) and Fe_2_O_3_ (2.6 %)] were used (Fig. 1). Hap tablets (5 mm diameter, 2–3 mm height), were supplied by the Institute of Silica Materials, Riga Technical University, Latvia and prepared by dry pressings of Hap nanopowders (4–10 nm) (Aronov et al. 2007). Chamotte tablets (15 mm diameter, 5 mm height) were prepared at the Institute of Fermentation Technology and Microbiology, Technical University of Lodz, Poland. They were made from water and chamotte fire clay (50–1,000 μm) (Boleslawiec Refractory Plant BZMO Ltd., Poland) in the ratio 1:2. The tablets were formed in special rubber forms, dried at 21 °C for 24 h and fired at 1,100 °C.

### Yeast strains and culture conditions

*Saccharomyces pastorianus* B4 (lager brewery strain), *S. cerevisiae* TT (ale brewery strain), *S. cerevisiae* Bc16a (distillery strain) and *Debaryomyces occidentalis* Y500/5 (amylolytic strain) from LOCK105 Culture Collection (Poland) were routinely maintained on wort agar slants (Merck). The yeasts were cultured in 50 mL wort broth (Merck) in 500 mL round bottom flasks at 30 °C on a rotary shaker at 220 rpm. For experimental purposes, after cultivation, the cells were washed twice with Ringer solution (Merck) by centrifugation (25 °C, 10 min, 2,000×*g*). The concentration of yeast cells was determined by counting them with a haemocytometer.

### Alcian blue retention assay

Adsorption of alcian blue by yeast cells was used as an indicator of the cell surface charge according to the method used by White and Walker (2011) with some modifications. The experiments were carried out in 2 mL silicon-coated tubes. Yeast cells after cultivation were centrifuged (25 °C, 10 min, 2,000×*g*), and resuspended in 0.02 M sodium acetate buffer (pH 4.0) to the concentration of 5 × 10^7^ cells mL^−1^. The yeast cell suspensions (1 mL) were resuspended in the 1.8 mL alcian blue dye buffer solution (50 mg L^−1^). The suspension was incubated for 30 min at 25 °C on an orbital shaker at 75 rpm, then centrifuged and the free dye remaining in the supernatant was determined by OD at 615 nm. The concentration of alcian blue was measured by reference to an alcian blue standard curve prepared from the original dye/buffer solution. Alcian blue retention was expressed as mg of alcian blue per 5 × 10^7^ cells. All yeast strains, in different physiological states, were tested in triplicate.

### Evaluation of cell surface hydrophobicity

Yeast hydrophobicity was measured by assaying the partitioning of yeast cells between the aqueous and hydrophobic hydrocarbon phase after vigorous mixing (Bester et al. 2012). Yeast cells after cultivation were centrifuged (25 °C, 10 min, 2,000×*g*), washed twice and resuspended in the 0.2 M sodium phosphate buffer (pH 7.0) to the concentration of 1 × 10^8^ cells mL^−1^. The volume of 20 mL of yeast suspension was placed in a separatory funnel, mixed vigorously (0.5 min) with 5 mL of xylene and left to stand for 30 min, whereupon the number of yeast cells in the aqueous phase was measured by counting them with a haemocytometer.

### Yeast immobilization

Immobilization of four yeast strains was conducted on two types of carriers. Because the physiological state of yeast cells may directly affect the adhesion efficiency, three different ways, including two phases of growth (logarithmic, stationary) and starving cells (cultivated for 24 h in Ringer solution) were applied. Yeast cells after cultivation were washed with Ringer solution and resuspended to the densities 1 × 10^7^, 5 × 10^7^ and 1 × 10^8^ cells mL^−1^. For each adhesion experiment, 10 sterile tablets of each carrier were used. The carriers were incubated with yeast suspension in the proper medium (10 mL) at 30 °C for 24 h with gentle agitation (75 rpm). In the case of starved cells, initially suspended in Ringer’s solution, adhesion was conducted in (1) wort broth, (2) minimal medium M_o_ [(NH_4_)_2_ SO_4_ 3 g L^−1^; KH_2_PO_4_ 1 g L^−1^, MgSO_4_·7H_2_O·0.5 g L^−1^, yeast extract (Difco) 0.5 g L^−1^, CaCO_3_·3 g L^−1^] with maltose (1 %) (brewery and distillery yeasts), or (3) minimal medium M_o_ with starch (1 %) (amylolytic strain). Following this, the carriers of each type were tested for adhesion.

### Control of the adhesion process

The replicate samples were prepared in order to assess adhesion with the use of two methods: (1) qualitative microscopic analysis by methylene blue-staining of cells in situ and (2) direct enumeration of cells following the removal of the cells from the carrier. For the qualitative and quantitative analysis, 10 tablets of each carrier from each experimental sample were analyzed.

For the qualitative assessment of the degree of immobilization, the adhered cells were visualized by fixing the carrier with 1:1 ethanol/diethyl ether, staining with methylene blue (0.01 %) and rinsing with water (White and Walker 2011). The images of the stained cells on the carrier surface were obtained using a light microscope Olympus BX41 with top illumination of the carrier surface with an external lamp. For each of the five carriers, 20 digital images with stained yeast cells were captured. Out of these images, 10 were randomly selected and analyzed. The level of cells adhesion was evaluated by comparison of the number of yeast cells on the images to an arbitrary scale of standards (0–5 points) and expressed as a sum of the collected points. Therefore, the maximum number of points for each carrier from each experimental sample was 250.

For the quantitative assessment of the degree of immobilization, Hap carriers were suspended in 1 mL of distilled water, whereas chamotte tablets were suspended in 1 mL of 5 % H_2_SO_4_. Then, tubes with the tablets and appropriate solutions were boiled for 2 min and vortexed for 15 min. After this time, the carriers were removed and the remaining solution was checked for the number of yeast cells. The number of cells released per tablet was determined by the fluorimetric method and DAPI (Sigma) staining. The fluorescence of the prepared samples was measured at the excitation wavelength 340 nm, and emission wavelength 455 nm, and controlled by the FLWINLAB software that analyzed and eliminated the background fluorescence. The concentration of yeast cells was determined by reference to a standard curve prepared from hot-denatured and DAPI-stained yeast cells. Calibration of the fluorimeter (Perkin Elmer LS 50B) using the prepared blank sample (water and DAPI) had been performed previously.

### Fermentations

The mini-fermentation trials were conducted in special 10 mL vials with a 2 mL medium sealed with a rubber bung and fermentation lock. The medium M_o_ with glucose (12 %) was used for all trials. The fermentation process was conducted, both for the adhered (on two carriers) and free yeast cells during 7 days at appropriate temperature (10 °C for lager yeast, 20 °C for ale yeast, 30 °C for distillery and amylolytic yeasts). The inoculum of free cells was the same as the amount of the immobilized yeast, determined earlier using the fluorimetric method.

### Control of the cell-carrier system stability

The cell loading of the ceramics was calculated by measuring the number of cells adhered on the carriers before and after fermentation. Additionally, the cell concentration in the liquid media after fermentation was measured using direct microscopic count.

### Measurement of the selected metabolic products of alcohol fermentation

The fermentation performance of the tested microorganisms was evaluated by GC analysis conducted for the samples of the fermented medium with Agilent 6890 gas chromatograph equipped with headspace autosampler, capillary Innowax column (60 m × 0.32 mm) and flame ionization detector (FID). Ethanol and 11 volatile flavor-active compounds—aliphatic higher alcohols (methanol, 1-propanol, 2-methyl-1-propanol 2 methyl-butanol, 3-methyl-butanol), esters (ethyl acetate, isopentyl acetate, ethyl butyrate, ethyl caproate, ethyl caprylate) and a carbonyl compound-acetaldehyde, were analyzed.

The experiments were carried out in triplicates, and the standard deviation was indicated.

## Results and discussion

### Immobilization of yeast cells on ceramic carriers

The microscopic observations carried out after 24 h of immobilization were performed following the process of methylene blue staining of the cells from the stationary phase of growth, immobilized on the carrier. It was found that the suspension density of cells from the stationary phase of growth had no significant effect on the adhesion process (Table 1). In most cases, slightly better efficiency of this process was observed for the suspensions containing 5 × 10^7^ cells per 1 mL. This density was chosen for the purpose of the subsequent experiments.

**Table 1:** Effect of the density of cell suspension on the adhesion process (qualitative assessment)

Strain	Carrier	Density of yeast cells (×10^7^ mL^−1^)		
		1	5	10
Bc16a	Hydroxylapatite	31.7 ± 3.1	45.3 ± 8.5	37.7 ± 5.0
	Chamotte	55.3 ± 2.1	85.7 ± 3.1	73.3 ± 2.5
Y 500/5	Hydroxylapatite	23.0 ± 2.6	29.7 ± 4.6	33.3 ± 5.7
	Chamotte	92.7 ± 4.0	84.3 ± 4.2	89.0 ± 7.2
TT	Hydroxylapatite	23.7 ± 4.6	23.6 ± 4.7	26.0 ± 4.3
	Chamotte	55.3 ± 3.8	60.3 ± 2.3	54.0 ± 2.6
B4	Hydroxylapatite	37.3 ± 4.5	46.7 ± 9.3	45.0 ± 3.6
	Chamotte	165.0 ± 13.3	178.0 ± 6.3	171.0 ± 6.1

**Fig. 2:**
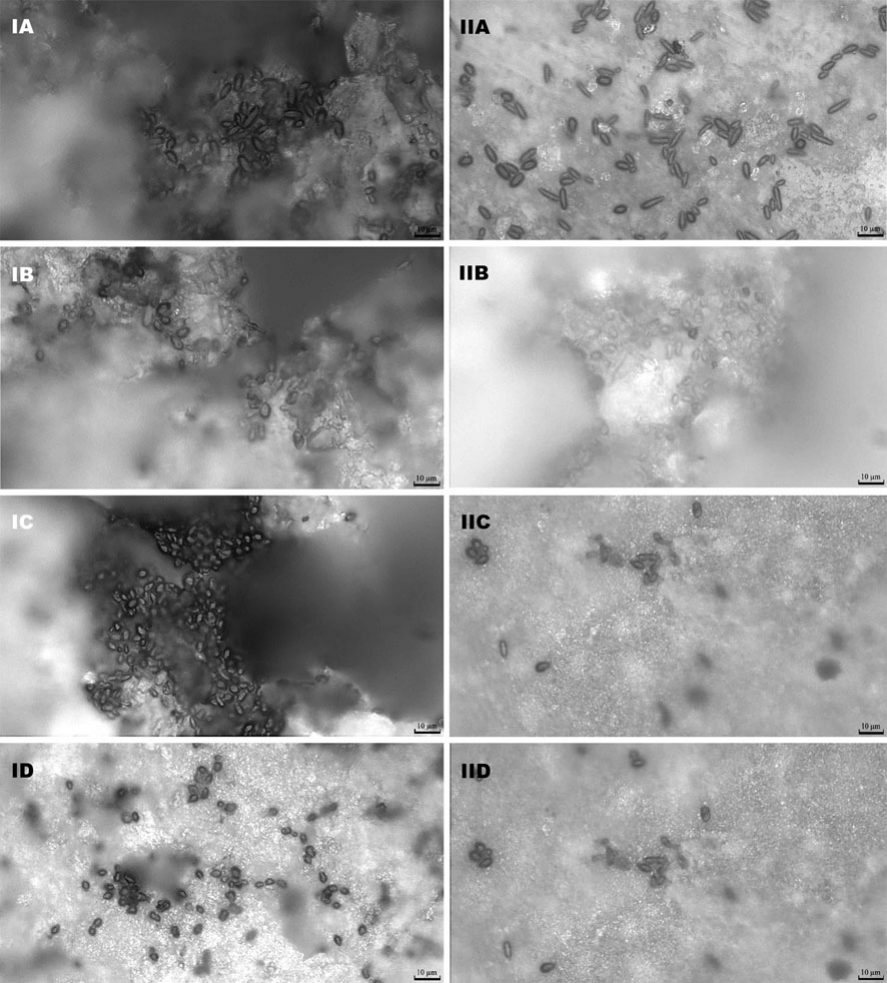
The cells of: **a** *Saccharomyces cerevisiae* TT, **b** *S. pastorianus* B4, **c** *S. cerevisiae* Bc16a, **d** *Debaryomyces occidentalis* Y500/5 on: I—chamotte, II—hydroxylapatite after adhesion and methylene blue-staining

Several important factors are known to influence the attachment of microbial cells to solid carrier materials. One of them is the physical structure of the carrier, mainly the pores and their distribution, which has a significant effect on the degree of yeast cell immobilization (Gallardo-Moreno et al. 2004; Pereira et al. 2000). Comparing the hydroxylapatite and chamotte carriers after 24 h of immobilization, significantly better results were obtained in the case of immobilization conducted on the more porous chamotte tablets, but formation of a typical three-dimensional structure was not observed (Table 2, Fig. 2).

**Table 2:** Yeast adhesion to hydroxylapatite and chamotte carriers

Strain	Carrier	Number of cells (×10^7^ per carrier)	Number of cells (×10^7^ per 100 mm^2^)
Bc16a	Hydroxylapatite	0.10 ± 0.05	0.63 ± 0.32
	Chamotte	1.71 ± 0.47	1.71 ± 0.47
Y 500/5	Hydroxylapatite	0.25 ± 0.09	1.60 ± 0.57
	Chamotte	1.23 ± 0.29	1.23 ± 0.29
TT	Hydroxylapatite	0.21 ± 0.04	1.32 ± 0.25
	Chamotte	1.95 ± 0.31	1.95 ± 0.31
B4	Hydroxylapatite	0.11 ± 0.04	0.69 ± 0.25
	Chamotte	4.15 ± 0.53	4.15 ± 0.53

The weak adhesion on the hydroxylapatite carriers may be explained by chemical instability of this type of carriers. Hydroxylapatite is a form of calcium phosphate, which has long been used for chromatographic separation of proteins and DNA or to adsorb biomolecules. Schröder et al. (2003) described that adsorption of proteins on hydroxylapatite surfaces is an easily reversible process and it depends on the concentration of PO_4_^3−^ ions and the pH level. Hydroxylapatite—calcium phosphate readily dissolves at lower pHs. Therefore, it is necessary to keep the pH level above 5.5 (Jang et al. 2000). During the growth and fermentation, there are produced numerous acidic metabolites, including acetaldehyde, ethyl acetate and others. Supplementation of the minimal medium M_o_ by CaCO_3_ prevents pH from decreasing, however, in wort broth there may occur uncontrolled decreasing of this parameter. This fact explains the better stability of the cell-Hap systems in minimal medium, and the lower stability in wort broth.

**Fig. 3:**
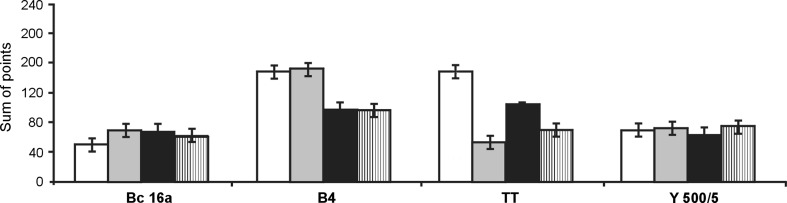
Qualitative assessment of the adhesion efficiency for yeast cells immobilized in different physiological states. (*open square*) logarithmic phase; (*grey shaded square*) stationary phase; (*black shaded square*) starving cells passaged in wort broth; (*line filled square*) starving cells passaged in minimal medium

Cell adhesion on hydroxylapatite was generally weak for all the yeast strains, therefore, only the results of the qualitative analysis of adhesion on chamotte carriers, with particular emphasis on the physiological state of yeast cells, are presented in Fig. 3. The maximum number of points for each carrier from each experimental sample was 250. In this study, the highest scores for yeast strains after 24 h of incubation were above 170 points.

The age of the cells or the growth phase may have a strong impact on the attachment properties of the cells. In the experiments carried out by Bowen et al. (2001), cell adhesion of *S. cerevisiae* brewing strain to the mica surface was significantly higher for the cells from the stationary growth phase compared to those from the logarithmic phase. It was also confirmed in our studies. A slightly higher immobilization efficiency in the case of cells from the stationary growth phase was observed for Bc16a, B4 and Y 500/5 strains, with the exception of TT that received the highest score in the logarithmic phase.

The cells from the log phase are usually characterized by greater sensitivity to changing environmental factors and lower resistance to stress, therefore, in further experiments we abandoned the cells derived from this phase of growth.

The limitation of the medium components is also one of the factors responsible for the synthesis of proteins classified as adhesins (Verstrepen and Klis 2006). The mechanism of adhesion of *S. cerevisiae* on glass and polymer carriers was identified by Guillemot et al. (2006) as substratum-dependent. In our experiments, for two strains *S. cerevisiae* TT and *D. occidentalis* Y 500/5, the nutrients limitation had a positive effect on the efficiency of adhesion.

The analysis of the results of our experiments demonstrates that the optimal conditions for yeast adhesion should be determined individually for each strain. The preferred phases of growth or physiological states for the tested yeast strains were as follows: *S. cerevisae* Bc16a—stationary phase of growth, *S. pastorianus* B4—stationary phase of growth, *S. cerevisae* TT—starving cells passaged in wort broth, *D. occidentalis* Y500/5—starving cells passaged in minimal medium M_o_ with starch.

Adhesion of yeast to a surface depends also on complex physicochemical interactions cell-surface-liquid phase. It was observed that localized positive charges of yeast cells could be very important in cell adhesion (Hermansson 1999). Microbial adhesion is also often associated with the overall surface hydrophobicity of microorganisms. It was claimed that increased cell-surface hydrophobicity favored cell adhesion (Aguedo et al. 2005; Liu et al. 2004). Adsorption of positively charged alcian blue to the cells is a typical electrostatic interaction. Yeast cells are predominantly characterized by the negative charge due to the presence of carboxyl, phosphoryl and hydroxyl groups. However, it was indicated that the overall cell wall charge was not the principal determinant in cell adhesion (White and Walker 2011).

**Fig. 4:**
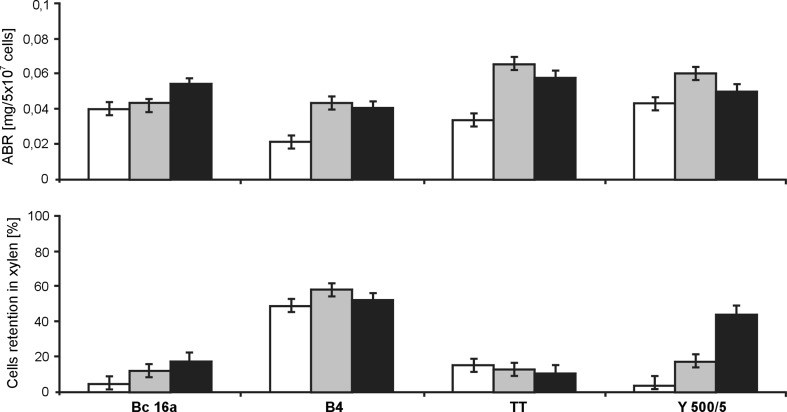
The surface charge and hydrophobicity of yeast cells in different physiological states. (*open square*) logarithmic phase; (*grey shaded square*) stationary phase;(*black shaded square*) starving cells

Our findings proved that cell surface hydrophobicity, as well as cell surface charge, differ significantly among all strains, indicating that these properties and their variations are characteristic for a particular strain. We observed that better adhesion occurred in the case of the brewery lager strain B4, with significantly greater surface hydrophobicity than in the case of other strains. This dependence was not observed for the alcian blue method (Fig. 4). The highest level of alcian blue retention was detected for the ale brewery strain TT and the amylolytic yeast Y500/5 from the stationary phase of growth. These conditions were different from the established optimal conditions for adhesion of these strains. On the basis of our results, we can assume that adhesion was rather controlled by the hydrophobic–hydrophilic surface properties than the electrostatic interactions.

**Fig. 5:**
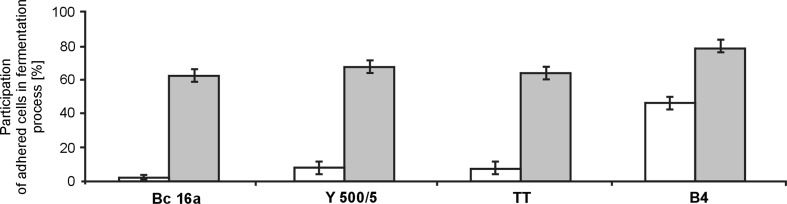
The stability of yeast-carrier systems during fermentation process. (*open square*) hydroxylapatite; (*grey shaded square*) chamotte

The carriers with immobilized cells were transferred into the fermentation medium M_o_ with 12 % of glucose. The cell-carrier systems were characterized by differential stability in the fermentation process (Fig. 5). Changing the culture medium to the fermentation medium did not cause destabilization of the cell-carrier systems. Nevertheless, the number of free cells in the medium was relatively high, as the immobilized cells accounted for approx. 20–70 % of all cells in the fermentation samples.

### Analysis of fermentation profiles

According to the literature, one of the most important limitations hampering widespread application of the immobilized yeast systems is the necessity to achieve the correct balance of volatile compounds to create an acceptable flavor profile of the product. Immobilized cells appear to have modified physiology compared to the physiology of free cells. The patterns of metabolites, such as high alcohols, esters, and carbonyl compounds usually change upon immobilization. The conditions that promote the yeast cell growth: high levels of nutrients (amino acids, oxygen, lipids), increased temperature, and agitation may stimulate the production of higher alcohols. On the other hand, conditions that restrict the yeast growth, e.g. lower temperature and higher CO_2_ pressure, reduce the extent of the higher alcohol production. Additionally, the mass transfer rates in the fermenting medium also influence higher alcohol synthesis. Since ethanol is the dominant alcohol in the fermentation process, ethyl acetate, produced from acethyl-CoA and ethanol, is the dominant ester. The anaerobic conditions and the absence of substantial levels of unsaturated fatty acids stimulate the formation of acetate esters. The most important carbonyl compounds formed in beverage fermentation are acetaldehyde, diacetyl and 2,3-pentanedione. The presence of diacetyl may contribute to the correct flavor, especially as regards cider and red wine. However, excessive production can lead to off-flavors, particularly in the case of beer (Nedovic et al. 2011).

Aromatic composition of the medium samples after fermentation with immobilized and free yeast strains is presented in Table 3. Immobilization affected significantly neither the production of high alcohols nor esters, especially since the variable results were obtained also for free cells in different trials. However, we observed a higher concentration of acetaldehyde in the fermentation medium in the case of the immobilized distillery yeast Bc16a on two kinds of carriers, and in the case of the top brewery strain TT on hydroxylapatite. These preliminary results for selected yeast strains need to be confirmed in industrial brewery wort taking into account different conditions of fermentation/maturation in order to obtain a final product with acceptable sensory characteristics.

**Table 3:** Fermentation profiles of free (F) and immobilized (I) yeast strains (mg L^−1^)

Strain	Acet-aldehyde		Ethyl acetate	Methanol	1-Propanol	Ethyl butyrate	2-Methyl-1-propanol	Izopentyl acetate	2-Methyl-1-butanol	Ethyl caproate	Ethyl caprylate
Hydroxylapatite											
TT	F	5.26 ± 0.51	26.92 ± 2.91	0.59 ± 0.06	26.24 ± 5.11	0.36 ± 0.04	14.25 ± 1.51	1.57 ± 0.14	25.12 ± 2.41	0.72 ± 0.06	13.95 ± 1.46
	I	25.42 ± 3.05	18.10 ± 1.97	1.46 ± 0.13	5.56 ± 5.23	0.31 ± 0.03	0.45 ± 0.50	0.00 ± 0.00	12.71 ± 1.53	0.05 ± 0.01	4.19 ± 0.51
B4	F	23.79 ± 2.39	3.54 ± 4.01	0.58 ± 0.08	0.00 ± 0.00	0.13 ± 0.01	10.96 ± 1.18	0.00 ± 0.00	8.14 ± 0.78	0.02 ± 0.01	0.11 ± 0.01
	I	12.99 ± 1.87	0.00 ± 0.00	1.87 ± 0.15	0.00 ± 0.00	0.02 ± 0.01	1.67 ± 0.19	0.00 ± 0.00	0.00 ± 0.00	0.00 ± 0.00	0.00 ± 0.00
Bc16a	F	2.16 ± 0.27	35.52 ± 2.72	0.61 ± 0.05	23.16 ± 2.23	0.46 ± 0.03	35.56 ± 3.02	2.00 ± 0.17	46.84 ± 4.39	0.56 ± 0.05	2.38 ± 0.27
	I	50.1 ± 5.63	30.32 ± 3.00	1.77 ± 0.19	25.92 ± 2.88	0.45 ± 0.03	35.11 ± 3.15	2.00 ± 0.22	39.41 ± 4.22	0.59 ± 0.04	7.41 ± 0.71
Y 500/5	F	1.48 ± 0.18	10.27 ± 1.1	0.00 ± 0.00	5.74 ± 0.55	0.24 ± 0.03	26.25 ± 2.92	4.16 ± 0.39	28.39 ± 3.01	0.52 ± 0.05	1.35 ± 0.12
	I	0.28 ± 0.07	58.15 ± 6.1	0.00 ± 0.00	4.74 ± 0.49	0.16 ± 0.02	45.21 ± 4.77	0.77 ± 0.08	16.85 ± 1.91	0.08 ± 0.01	0.18 ± 0.02
Chamotte											
TT	F	0.00 ± 0.00	33.59 ± 2.93	0.00 ± 0.00	44.06 ± 5.11	0.50 ± 0.06	25.49 ± 2.72	1.34 ± 0.12	34.45 ± 5.41	0.53 ± 0.04	9.53 ± 1.12
	I	0.41 ± 0.05	42.53 ± 5.97	1.74 ± 0.12	55.87 ± 5.23	0.41 ± 0.05	18.25 ± 2.17	3.16 ± 0.29	23.56 ± 2.13	0.69 ± 0.05	13.19 ± 1.51
B4	F	14.23 ± 1.31	29.11 ± 4.11	0.88 ± 0.09	10.56 ± 2.17	0.23 ± 0.01	32.44 ± 4.18	0.30 ± 0.02	21.06 ± 3.19	0.21 ± 0.01	1.16 ± 0.20
	I	29.15 ± 4.57	10.53 ± 2.45	1.68 ± 0.11	2.43 ± 0.33	0.13 ± 0.01	10.72 ± 1.19	0.00 ± 0.00	12.31 ± 1.51	0.06 ± 0.01	0.33 ± 0.03
Bc16a	F	4.30 ± 0.29	25.88 ± 2.22	0.67 ± 0.05	19.78 ± 2.18	0.55 ± 0.43	35.16 ± 4.11	1.39 ± 0.15	48.28 ± 4.81	0.43 ± 0.05	6.42 ± 0.07
	I	30.96 ± 5.12	38.67 ± 5.00	1.89 ± 0.21	25.92 ± 2.97	4.10 ± 0.63	38.92 ± 3.15	2.57 ± 0.21	42.24 ± 4.11	0.72 ± 0.10	6.32 ± 0.07
Y 500/5	F	6.95 ± 0.92	13.15 ± 2.1	0.40 ± 0.09	9.36 ± 1.11	0.25 ± 0.03	53.63 ± 4.91	0.92 ± 0.08	27.82 ± 3.18	0.11 ± 0.01	0.22 ± 0.02
	I	9.23 ± 1.27	11.03 ± 2.1	1.59 ± 0.13	8.24 ± 0.79	0.32 ± 0.04	59.28 ± 4.65	0.90 ± 0.78	34.12 ± 3.51	0.16 ± 0.01	0.31 ± 0.02

## Conclusions

Cell adhesion is the fundamental phenomenon that governs and describes bioengineering processes applying cell immobilization focusing on different biotechnological applications. Many issues should be taken into account, such as the physiological state of the yeast cells, product quality, safety and stability during processing as well as operating costs. Our research confirmed that the chemical character and microtopography of the support, nutrient availability and the physiological state of the cells are important in determining the adhesion processes. Selecting a suitable carrier and proper yeast strains for a stable cell-carrier system seems to be a very important step to useful immobilized cell technology. The main efforts should be concentrated on cheap, abundant, non-destructive and stable carriers, which will not influence the aroma profiles and specific taste of the final product. According to our results, we can formulate a conclusion that the conditions of immobilization and cell physiology are specific for each yeast strain, and the construction of efficient cell-carrier systems is possible only experimentally.
